# Dendrimers: A Themed Issue in Honor of Professor Donald A. Tomalia on the Occasion of His 85th Birthday, Recognizing His Outstanding Achievements in Advancing the Field of Dendrimers

**DOI:** 10.3390/pharmaceutics16121608

**Published:** 2024-12-17

**Authors:** Anne-Marie Caminade

**Affiliations:** 1 Laboratoire de Chimie de Coordination (LCC-CNRS) 205 Route de Narbonne, CEDEX 4, 31077 Toulouse, France; anne-marie.caminade@lcc-toulouse.fr; 2 LCC-CNRS, Université de Toulouse, CNRS, 31077 Toulouse, France

As the main pioneer in the field of dendrimers, a term that he coined in 1985 in reference to their tree-like structure, Prof. Donald A. Tomalia has inspired several generations of researchers worldwide. I am very happy to announce that our *Pharmaceutics* Special Issue callout dedicated to his 85th birthday was a great success, with 13 contributions, including several from the main players in the field and even a remarkable review by Prof. Donald A. Tomalia himself [1].

On the other hand, this launch also brings sadness, because the idea of this Special Issue was proposed by Prof. Zofia Urbanczyk-Lipkowska, who very unfortunately passed away on 22 January 2024. The announcement of her death was a great shock for me. I had known Zofia for a long time, as she had been involved in work on peptide dendrimers since 2003. We met several times at congresses about dendrimers, as well as in Poland, and we became friends. She really was a very kind person, as emphasized by the “In Memoriam” contribution of Donald A. Tomalia in the paper that he wrote for this Special Issue [1]. Rest in peace, Zofia—your friends will never forget you.

This Special Issue of *Pharmaceutics* contains 13 papers, 4 reviews and 9 research articles, that illustrate the diversity of dendrimers, dendrons, and dendritic structures. Their diverse potential uses, such as in pharmaceutical sciences, against cancers, in macular degeneration in the eyes, for vaccines, in drug delivery, and in diagnosis, are emphasized. This Special Issue provides an up-to-date overview and the latest results in relation to dendrimers as bio-tools.

The review written by D.A. Tomalia [1] is an important and thought-provoking contribution, reflecting on the beginning, past, present, and future of dendrimers in pharmaceutics, medicine, and the life sciences, told over 40 pages. It begins with an historical perspective on the beginnings of dendrimers, in which D.A. Tomalia played a major role, in particular with the elaboration of the PAMAM (polyamidoamine) dendrimers and the creation of the name dendrimers. The review continues with the concept of critical nanoscale design parameters (CNDPs), which he elaborated to classify and predict the characteristics of nanoparticles, including dendrimers. The last part explores anticipated new applications for dendrimers and predicts the roles that they could be playing in ten years’ time (2034) as advanced nano-pharmaceuticals for drug delivery and imaging.

The review by D. Astruc et al. [2] concerns ferrocene-based drugs, with an emphasis on their delivery using nanosystems, micelles, metal–organic frameworks (MOFs), polymers, and dendrimers. One of the main advantages of ferrocenes in pharmacy is that they induce the production of reactive oxygen species (ROS). The main ROS generated by ferrocenes is the OH^.^ radical, produced by Fenton catalysis, which is the reaction of Fe^2+^ with H_2_O_2_ and is particularly suitable for killing cancer cells.

The review by A.M. Caminade et al. [3] is about the biological properties of dendritic structures functionalized with boron clusters. These compounds are potentially useful in the treatment of cancers using boron neutron capture therapy (BNCT), the efficiency of which is directly correlated with the number of boron atoms. The BNCT method starts from ^10^B, a nonradioactive natural element, which is irradiated with low-energy thermal neutrons to generate high-linear-energy transfer particles that are suitable for killing cancer cells. Boronated dendrimers increased the mean survival time in BNCT in vivo experiments.

The review by V. Ceña et al. [4] concerns the in vivo applications of dendrimers as drug carriers. It describes, firstly, experiments carried out on cancer cells, and then in vivo experiments, mainly in mice, in the treatment of diverse diseases, such as cancers, neurodegenerative diseases, infections, and ocular diseases, but also as diagnostic agents. The very few clinical trials carried out with dendrimer formulations up until now are also presented.

The quite large paper by D. Weissman, V. Percec et al. [5] present an extensive paper comprising 50 pages and 166 pages of Supporting Information. It concerns the synthesis of large libraries of amphiphilic Janus dendrimers, derived from renewable plant phenolic acids, and their use in the targeted delivery of mRNA. The screening of these libraries using the CNDP principles demonstrated that the precise location of the functional groups can induce the targeting of precise organs, including the liver, spleen, lymph nodes, and lungs. These Janus dendrimers can also facilitate the handling and storage of vaccines, such as COVID-19 mRNA vaccines.

The paper by M.C. Daniel et al. [6] concerns the use of PPI (polypropylene imine) dendrons equipped with a disulfide (thioctic acid) at the focal point for their attachment to gold nanoparticles. The dendrons are functionalized on their surface either with doxorubicin or with azides. Both dendrons are then linked to gold nanoparticles (NPs), and a few azides are reacted with an EphA2-targeting antibody fragment to target the prostate cancer cell PC3. These dendronized gold NPs were tested and were found to be very efficient (IC_50_ = 0.9 nM) against PC3 cancer cells.

The paper by R.M. Kannan et al. [7] describes the grafting of some integrin-binding ALG-1001 peptides to generation 6 hydroxyl-terminated PAMAM dendrimers to provide a potential alternative treatment against wet age-related macular degeneration in the eyes. This disease is characterized, in particular, by the abnormal growth of blood vessels. This dendrimer has been found to be suitable for protecting ALG-1001 against degradation by proteinases and induces significant reductions in CNV lesion areas (choroidal neovascularization or the creation of new blood vessels) in vivo in rat eyes.

The paper by E. Mohammadifar, R. Haag et al. [8] concerns both a polyglycerol dendron G2 functionalized with cholesterol at the core and dendritic structures consisting of a block copolymer of the type poly(G1-polyglycerol dendron methacrylate)-block-poly(cholesterol methacrylate). Both the dendron and the dendronized copolymer form micelles in water. Cell viability studies on A549 cells demonstrated that the dendron is toxic, but not the copolymer. The encapsulation of doxorubicin (DOX) by the copolymer induced the accumulation of DOX in the cell nucleus.

In the study by M. Valiente, R. Gomez et al. [9], cholesterol was also grafted to the core of carbosilane dendrons (G1 to G3) equipped with ammonium terminal groups. Another series of dendrons, with d-α-tocopherol (vitamin E) as the focal point, were also synthesized. The cell toxicity was in the range of 10–20 μM for both families. They formed micelles in water, which were used for the encapsulation of drugs (such as ibuprofen, lidocaine, procaine, or diclofenac), the release of which was studied.

The paper by S. Garcia-Gallego et al. [10] concerns small carbosilane dendrons and dendrimers bearing alkene functions and their use as cross-linkers by “thiol-ene” coupling with dithiol polymers to produce hydrogels. The nature of the hydrogel depends on the type of polymers, affording either non-swelling (propylene glycol and Pluronics L31 and L61) or high-swelling (polyethylene glycol and Pluronics L35) hydrogels with thermo-responsive behavior. These hydrogels are suitable for drug release, as illustrated with caffeic acid.

The paper by E. Laurini, S. Pricl et al. [11] describes a PAMAM dendron with two alkyl chains as a tail and primary amines as terminal functions, and its association with human serum albumin (HAS), which plays a crucial role in pharmacokinetics. This interaction is studied both experimentally (via fluorescence spectroscopy, circular dichroism, calorimetry, dynamic light scattering, and zeta potential) and theoretically (through computational simulations at the atomistic level using the AMBER22 main program CPPTRA).

The paper by C. Kojima et al. [12] concerns both PAMAM dendrimers and dendri-graft polylysine (DGL), functionalized with phenylalanine (Phe), and their association with Jurkat cells, a T-cell model. The strength of the association is higher with functionalized DGL than with PAMAM, but the functionalized DGL is insoluble in water, and thus unsuitable as a drug carrier. Model drugs (protoporphyrin IX and paclitaxel) loaded in PAMAM-Phe exhibit similar toxicities against Jurkat cells than free drugs.

The paper by M. I. Montañez, Y. Vida et al. [13] is also about PAMAM dendrimers, exploring their use in the decoration of silica NPs and when functionalized with amoxicillin. Free amoxicillin is used in routine diagnosis, as it is assumed to conjugate to a carrier present in blood, but it does not afford information on the size and composition of these conjugates. The nanocomposite can specifically activate basophils more efficiently than free amoxicillin, making it an accurate tool in the diagnosis of allergy to betalactam antibiotics.

To conclude, from myself and from all the contributors to this Special Issue of *Pharmaceutics*, Don, I wish you a happy birthday and a long and happy life.
